# Synaptopathy as a Mechanism for Age-Related Vestibular Dysfunction in Mice

**DOI:** 10.3389/fnagi.2019.00156

**Published:** 2019-06-26

**Authors:** Guoqiang Wan, Lingchao Ji, Thomas Schrepfer, Sihao Gong, Guo-Peng Wang, Gabriel Corfas

**Affiliations:** ^1^Kresge Hearing Research Institute, Department of Otolaryngology—Head and Neck Surgery, University of Michigan, Ann Arbor, MI, United States; ^2^MOE Key Laboratory of Model Animals for Disease Study, Model Animal Research Center, Nanjing University, Nanjing, China; ^3^Department of Otolaryngology, Chinese PLA General Hospital, Beijing, China

**Keywords:** inner ear, utricle, vestibular dysfunction, hair cells, ribbon synapses, synaptopathy, calyx

## Abstract

Age-related decline of inner ear function contributes to both hearing loss and balance disorders, which lead to impaired quality of life and falls that can result in injury and even death. The cellular mechanisms responsible for the ear’s functional decline have been controversial, but hair cell loss has been considered the key cause for a long time. However, recent studies showed that in the cochlea, loss of inner hair cell (IHC) synapses precedes hair cell or neuronal loss, and this synaptopathy is an early step in the functional decline. Whether a similar process occurs in the vestibular organ, its timing and its relationship to organ dysfunction remained unknown. We compared the time course of age-related deterioration in vestibular and cochlear functions in mice as well as characterized the age-associated changes in their utricles at the histological level. We found that in the mouse, as in humans, age-related decline in vestibular evoked potentials (VsEPs) occurs later than hearing loss. As in the cochlea, deterioration of VsEPs correlates with the loss of utricular ribbon synapses but not hair cells or neuronal cell bodies. Furthermore, the age-related synaptic loss is restricted to calyceal innervations in the utricular extrastriolar region. Hence, our findings suggest that loss of extrastriolar calyceal synapses has a key role in age-related vestibular dysfunction (ARVD).

## Introduction

Dizziness, vertigo, and imbalance are among the most common complaints in people over 65 years of age (Dunn et al., 1992; Whitney et al., 2002). Fall-related injuries are the number one cause of injuries and deaths from injury among older Americans (cdc)^1^, with an estimated mortality rate of 12% (Dunn et al., 1992). The stability of posture and gaze during standing and walking is maintained by the rapid processing of vestibular, visual, and somatosensory inputs in the central nervous system, followed by outputs to the musculoskeletal and visual systems (Iwasaki and Yamasoba, 2015). As all neural circuits contributing to these sensory inputs can deteriorate during aging (Barin and Dodson, 2011), the underlying causes of dizziness and vertigo vary widely among the elderly, but peripheral vestibular disorders are the most common cause (Davis, 1994; Karatas, 2008). Identifying the cellular basis for this age-related vestibular dysfunction (ARVD) would help characterize its mechanisms and therefore provide molecular targets for future therapies.

The vestibular end organs consist of three semi-circular canals and two otolith organs, the utricle and saccule. Each canal and otolith organ have a small sensory epithelium populated by hair cells, i.e., cristae in the canals, maculae in the utricle and saccule. Hair cells in the cristae respond to rotatory or angular movements of the head; those in the maculae are activated by gravity or linear acceleration (Khan and Chang, 2013). The sensory epithelia of the utricle and saccule are heterogeneous in terms of type and distribution of hair cells and their neuronal innervations (Eatock and Songer, 2011). Based on morphology, macular hair cells are classified into types I and II. Type I hair cells appear globular or flask-shaped and are innervated by a single calyceal afferent terminal that engulfs their basolateral wall. Type II hair cells are cylindrical and innervated by multiple small afferent bouton terminals. Both types of hair cells are differentially distributed in sub-regions of the otolith organ epithelia, i.e., the central striola and peripheral extrastriolar region (Burns and Stone, 2017).

Like for age-related hearing loss (Yamasoba et al., 2013), it has been hypothesized that vestibular hair cell loss is a major cause of ARVD (Brosel et al., 2016). For instance, age-related hair cell loss in the cristae has been documented in rodents and humans (Rosenhall, 1973; Nakayama et al., 1994), a process that could contribute to imbalance in the elderly. In contrast, age-related hair cell loss in the otolith organs appears to be much milder (Nakayama et al., 1994; Gopen et al., 2003). Yet, ocular vestibular evoked myogenic potentials (oVEMPs) in humans, which reflect utricular function (Iwasaki et al., 2009), clearly show age-dependent decline in amplitude and increase in latency (Iwasaki et al., 2008; Agrawal et al., 2012). Such age-related utricular dysfunction is consistent with increased tendencies toward tilting and imbalance in the elderly; however, it is unknown which cells or structures undergo age-related deterioration in the utricular system.

To gain insights into the cellular basis of otolith organ dysfunction, we investigated the impact of aging on the integrity of the utricular system in mice by testing its function and structure at ages ranging from 2 to 24 months. Recordings of vestibular evoked potentials (VsEPs) show that defects are apparent only by 24 months, much later than the onset of hearing loss. The alterations in VsEPs correlate with significant loss of utricular ribbon synapses, which occurs in the absence of loss of hair cells or neurons. Remarkably, the synaptic loss is restricted to calyceal innervations within the extrastriolar region. Together, our results show that the onset of otolith organ dysfunction in mice, as in humans, occurs much later than hearing loss, that synaptopathy might be a key contributor to this decline, and that extrastriolar calyceal terminals might be especially susceptible to age.

## Materials and Methods

### Animals

Wild-type FVB/N mice of both sexes were tested at 2, 5, 9, 18, and 24 months of age. All animal procedures were approved by the Institutional Animal Care and Use Committee of the University of Michigan.

### Physiological Tests

Mice were anesthetized with xylazine (10 mg/kg, i.p.) and ketamine (100 mg/kg, i.p.) prior to inner ear physiological tests, including VsEP, auditory brainstem response (ABR), and distortion-product otoacoustic emission (DPOAE). For VsEP and ABR recordings, three needle electrodes were placed into the skin: one at the dorsal midline close to the neural crest, one behind the left pinna and one at the base of the tail for grounding. For VsEP recording, mice were positioned on their backs with the head coupled securely to a shaker platform. Stimuli consisting of linear acceleration ramps with 2 ms in duration were applied in the earth-vertical axis at 17/s with alternating stimulus polarity. An accelerometer mounted near the head was used to calibrate the resultant jerk, which was expressed in dB re 1.0 g/ms. Electrophysiological activity was amplified (10,000×), filtered (0.3–3 kHz), and digitized (125 kHz), 1,024 responses were averaged at each stimulus level. We collected an intensity series in 2.5 dB steps encompassing −20 dB to 0 dB re 1.0 g/ms. To measure auditory function, ABR potentials were evoked with 5 ms tone pips (0.5 ms rise-fall, with a cos2 envelope, at 33/s) delivered to the eardrum at log-spaced frequencies from 5.6 to 32 kHz. The response was amplified (10,000×) and filtered (0.3–3 kHz) with an analog-to-digital board in a PC-based data-acquisition system. Sound pressure level (SPL) was raised in 5 dB steps from 10 to 80 dB. At each level, 1,024 responses were averaged (with stimulus polarity alternated) after “artifact rejection.” The DPOAE in response to two primary tones with frequencies f1 and f2 was recorded at the third frequency (2 × f1) − f2, with f2/f1 = 1.2, and the f2 level 10 dB lower than the f1 level. Ear-canal sound pressure was amplified and digitally sampled at 4 μs intervals. DPOAE threshold was defined as the f1 level required to evoke a response at −10 dB SPL.

### Immunofluorescence

Mice were perfused intracardially with 4% paraformaldehyde in 0.1 M phosphate buffer. Temporal bones were post-fixed in 4% paraformaldehyde in 0.1 M phosphate buffer for 2 h at room temperature and decalcified in 5% EDTA. For cryo-section analysis, the temporal bones were embedded and frozen in OCT media (Sakura Finetek, Torrance, CA, USA), followed by cryosectioning at 14 μm thickness. The sections were dried at 37°C for 1 h, rinsed with phosphate-buffered saline (PBS) before subsequent immunostaining analysis. For wholemount immunostaining analysis, utricles were subsequently micro-dissected and permeabilized by freeze-thawing in 30% sucrose. The micro-dissected utricles were blocked in 5% normal horse serum with 1% Triton X-100 in PBS for 1 h, followed by incubation in primary antibodies (diluted in blocking buffer) at 25°C for 16 h. The primary antibodies used in this study were anti-myosin VIIa (rabbit anti-MyoVIIa, 25-6790, Proteus Biosciences, 1:500), anti-C-terminal binding protein 2 (mouse anti-CtBP2 IgG1, 612044, BD Biosciences, 1:200), anti-glutamate receptor 2 (mouse anti-GluA2 IgG2a, MAB397, Millipore, 1:2,000), anti-tenascin C (rabbit anti-tenascin, AB19013, Millipore, 1:500), anti-secreted phosphoprotein 1 (or osteopontin; goat anti-Spp1, AF808, RnD Systems, 1:500), anti-βIII-tubulin (mouse Tuj1 IgG2a, MMS-435P, BioLegend, 1:1,000) and anti-sox2 (goat anti-sox2, sc-17320, Santa Cruz Biotechnology). Utricles were then incubated with appropriate Alexa Fluor-conjugated fluorescent secondary antibodies (Thermo Fisher Scientific; 1:500 in blocking buffer) for 1 h at room temperature and mounted on microscope slides in ProLong^®^ Gold Antifade Mountant (P36930, Thermo Fisher Scientific).

### Confocal Imaging and Analyses

Confocal z-stacks of the utricle sensory epithelia were taken using a Leica SP8 microscope equipped with either 40× (0.75× optical zoom) or 63× (5× optical zoom) oil-immersion lens. The number of hair cells and the size of sensory epithelium were determined based on the Myo7a immunofluorescence. The numbers of type I hair cells were determined by immunofluorescence of secreted phosphoprotein 1 (Spp1), which is also known as osteopontin (Opn). The numbers of calyces were determined by immunofluorescence of tenascin C (Tnc). For synaptic counts, confocal image stacks (Ctbp2/GluA2 and Ctbp2/Tnc) were imported to Amira software (Visage Imaging, San Diego, CA, USA), which produced three-dimensional (3D) renderings of each confocal z-stack using the “connected components” feature. Ctbp2 puncta in each image stack were then captured and counted automatically. To assess the appositions of Ctbp2 with GluA2 puncta (putative ribbon synapses) or Ctbp2 puncta with Tnc (calyceal synaptic ribbons), the z-stacks were re-imaged using custom software that computed and displayed the x-y projection of the voxel space within 0.5 μm of the center of each puncta, as identified by Amira analysis. The number of juxtaposed Ctbp2/GluA2 puncta or Ctbp2/Tnc was visualized and counted from these miniature image arrays. For each utricle, three areas (36.9 μm × 36.9 μm) each of the striolar and extrastriolar regions were imaged and averaged as one data point for each region (striola and extrastriola). The number of utricles analyzed at each age was depicted in the figures and legends.

### Plastic Sections

Mice were perfused intracardially with 4% paraformaldehyde in 0.1 M phosphate buffer. Vestibular ganglia were extracted and post-fixed with 1.5% paraformaldehyde and 2.5% glutaraldehyde. The vestibular ganglia were then osmicated in 1% osmium tetroxide, decalcified in 5% EDTA, dehydrated, and embedded in araldite. The embedded vestibular ganglia were hardened at 60°C for 5 days and semi-thin (1 μm) sectioned using a Leica UC6 ultramicrotome. The semi-thin sections were mounted on microscope slides in Permount (Fisher Scientific) and the vestibular ganglion cell bodies imaged under light microscope using 63× DIC optics. Three sections spanning 60 μm each were imaged, counted, and averaged. The number of vestibular ganglia analyzed at each age was depicted in the figure and legend.

### Statistical Analysis

Statistical tests were performed using Graphpad Prism 6 (Graphpad Software Inc., La Jolla, CA, USA). Statistical differences in auditory physiology (ABR, DPOAE) were analyzed using two-way ANOVA comparing to the 2-month group. The size of sensory epithelium and the density of hair cell, ribbon synapse, calyx, and vestibular ganglion were analyzed using the one-way ANOVA comparing to the 2-month group, followed by Dunn’s multiple comparisons test.

## Results

### Age-Related Auditory and Vestibular Dysfunction in Mice Have Different Onset

To determine the effects of aging on vestibular function, we studied FVB/N mice at the ages of 2, 5, 9, 18, and 24 months. VsEPs, the summed activity of the utricular and saccular afferent pathways to head motions (Jones and Jones, 1999), were used to evaluate the function of the vestibular otolith organs. In the same mice, we also evaluated hearing to compare the effects of aging on both aspects of inner ear function.

**Figure 1:**
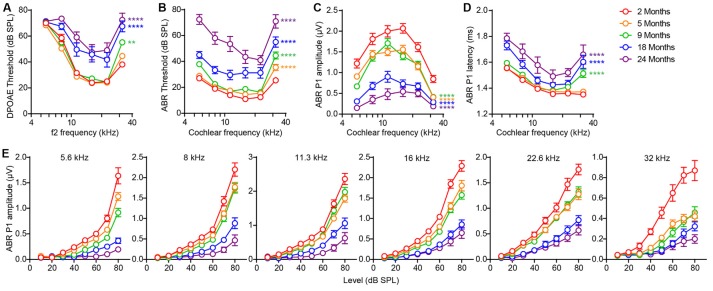
Signs of age-related hearing loss in FVB/N mice are detectable at 5 months of age. **(A)** Distortion-product otoacoustic emission (DPOAE) threshold, **(B)** auditory brainstem response (ABR) threshold, **(C)** ABR peak 1 amplitude, and **(D)** latency were measured on 2 (*n* = 10), 5 (*n* = 12), 9 (*n* = 12), 18 (*n* = 12) and 24 (*n* = 10) month old FVB/N mice. ABR peak 1 latency was analyzed at 80 dB sound pressure level (SPL) and amplitude analyzed by averaging 70 and 80 dB SPL. **p < 0.01 and ****p < 0.0001 by two-way ANOVA comparing to 2-month-old mice. **(E)** ABR peak 1 amplitude was plotted against the acoustic stimulus level (10–80 dB SPL) at 5.6, 8, 11.3, 16, 22.6 and 32 kHz. Input-output curves of ABR P1 amplitudes from 5, 9, 18 and 24-month-old mice were significantly different from that of 2-month-old mice at all frequencies tested (*p* < 0.0001 by two-way ANOVA).

Outer hair cell (OHC) dysfunction, measured as elevation of thresholds for DPOAEs, was first evident at high frequency (32 kHz) by 9 months and extended to all frequencies by 18 months (Figure 1A; 9 vs. 2 months, *F*_(1,120)_ = 10.91, *p* = 0.0013; 18 vs. 2 months, *F*_(1,120)_ = 60.36, *p* < 0.0001; 24 vs. 2 months, *F*_(1,108)_ = 124.1, *p* < 0.0001; all by two-way ANOVA). Thresholds for ABRs, the summed activity of the auditory afferent pathway activated by inner hair cells (IHCs) were elevated at high frequency (32 kHz) even earlier, at 5 months, and extended to all frequencies by 18 months (Figure 1B; 5 vs. 2 months, *F*_(1,120)_ = 30.05, *p* < 0.0001; 9 vs. 2 months, *F*_(1,120)_ = 98.09, *p* < 0.0001; 18 vs. 2 months, *F*_(1,120)_ = 141.9, *p* < 0.0001; 24 vs. 2 months, *F*_(1,108)_ = 374.4, *p* < 0.0001; all by two-way ANOVA). Interestingly, suprathreshold amplitudes of the first peak of the ABR wave (peak 1), which reflect the size of the sound-evoked spiral ganglion neuron compound action potential and are influenced by the density of IHC synapses (Buchwald and Huang, 1975; Wan et al., 2014), showed significant reductions at all frequencies by 5 months (Figures 1C,E; 5 vs. 2 months, *F*_(1,120)_ = 61.24, *p* < 0.0001; 9 vs. 2 months, *F*_(1,120)_ = 68.43, *p* < 0.0001; 18 vs. 2 months, *F*_(1,120)_ = 298.9, *p* < 0.0001; 24 vs. 2 months, *F*_(1,108)_ = 414.9, *p* < 0.0001; all by two-way ANOVA), indicative of hidden hearing loss (Sergeyenko et al., 2013). Together, these results demonstrate that FVB/N mice develop hidden hearing loss by 5 months that progresses with aging, resulting in overt hearing loss by 18 months, i.e., significant elevation of DPOAE and ABR thresholds at all frequencies (Figures 1A,B). Furthermore, suprathreshold ABR peak 1 latencies were significantly increased by 9 months (Figure 1D; 9 vs. 2 months, *F*_(1,120)_ = 34.40, *p* < 0.0001; 18 vs. 2 months, *F*_(1,120)_ = 65.90, *p* < 0.0001; 24 vs. 2 months, *F*_(1,101)_ = 97.04, *p* < 0.0001; all by two-way ANOVA).

**Figure 2:**
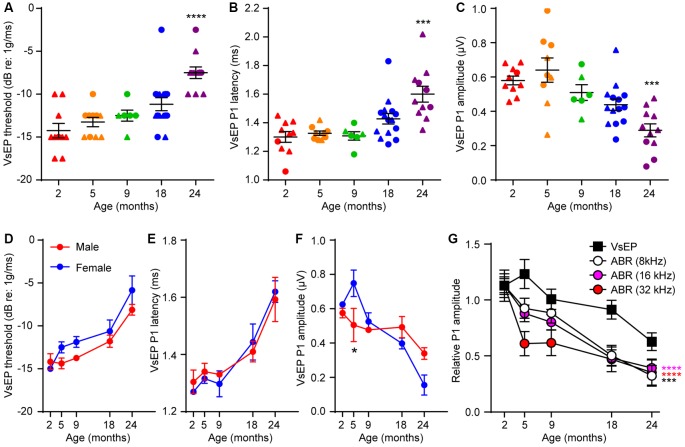
Age-related defects in vestibular evoked potentials (VsEPs) are seen only in 24 month-old-mice. **(A)** VsEP threshold, **(B)** VsEP peak 1 latency and **(C)** VsEP peak 1 amplitude at 2.5 dB re 1 g/ms were measured on 2 (*n* = 10), 5 (*n* = 10), 9 (*n* = 6), 18 (*n* = 15) and 24 (*n* = 11) month old FVB/N mice. Data from males and females are presented as triangles and circles respectively. ***p < 0.001 and ****p < 0.0001 by one-way ANOVA comparing to 2-month-old mice followed by Dunn’s post-test. **(D)** VsEP threshold, **(E)** VsEP peak 1 latency and **(F)** VsEP peak 1 amplitude at 2.5 dB re 1 g/ms of either male or female mice were analyzed separately. The numbers of males and females at each point are 9 and 1 (2 months), 4 and 6 (5 months), 7 and 8 (18 months), 2 and 4 (9 months), 8 and 3 (24 months) respectively. *p = 0.03 by two-way ANOVA. **(G)** Comparison of the time course of age-related changes in VsEP peak 1 amplitude and ABR peak 1 amplitude. VsEP and ABR (8, 16, 32 kHz) peak 1 amplitude was normalized to data from 2-month-old mice. The relative amplitude was plotted against age of the mice. ***p < 0.001 and ****p < 0.0001 by two-way ANOVA comparing to changes in VsEP amplitude.

In contrast to the timing of hearing loss, VsEP threshold elevation was only seen at 24 months (Figure 2A; Kruskal–Wallis statistic = 27.55, *p* < 0.0001, one-way ANOVA followed by Dunn’s multiple comparisons test). Furthermore, 24-month-old mice exhibited increased VsEP peak 1 latencies (Figure 2B; Kruskal–Wallis statistic = 24.14, *p* = 0.0003, one-way ANOVA followed by Dunn’s multiple comparisons test) and reduced VsEP peak 1 amplitudes (Figure 2C; Kruskal–Wallis statistic = 24.65, *p* = 0.0002, one-way ANOVA followed by Dunn’s multiple comparisons test). We found no significant difference in VsEPs between males and females (Figures 2D–F), except for slightly higher VsEP P1 amplitudes in female mice at 5 months (Figure 2F; *t* = 2.751, *p* = 0.03 by two-way ANOVA). However, given the small samples size for each sex and that we see no difference at any other timepoint, the difference at 5 months most probably reflects the inherent variability in the recordings or the aging process. Comparison of the peak 1 amplitudes of both ABRs and VsEPs over 24 months shows that the rate of age-related decline is slower for VsEPs (Figure 2G; ABR (8 kHz) vs. VsEP, *F*_(1,98)_ = 14.27, *p* = 0.0003; ABR (16 kHz) vs. VsEP, *F*_(1,98)_ = 20.31, *p* < 0.0001; ABR (32 kHz) vs. VsEP, *F*_(1,98)_ = 25.09, *p* < 0.0001; all by two-way ANOVA).

### Hair Cell Density Remains Stable in the Aging Utricular Macula

**Figure 3:**
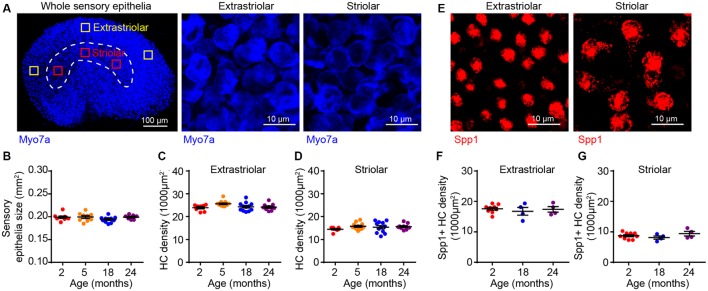
Utricular size and density of utricular hair cells do not change with aging. **(A)** Myo7a immunofluorescence images showing the entire sensory epithelium of an utricle. Squares represent high magnification sampling of extrastriolar and striolar (dotted lines) areas. **(B)** The size of utricle sensory epithelium in 2 (*n* = 9), 5 (*n* = 9), 18 (*n* = 11) and 24 (*n* = 7) month old FVB/N mice. Density of utricular hair cells in extrastriolar area **(C)** and striolar area **(D)** in 2 (*n* = 8), 5 (*n* = 12), 18 (*n* = 12) and 24 (*n* = 9) month old FVB/N mice. **(E)** Representative confocal images of Spp1+ type I hair cells in extrastriolar and striolar regions. **(F)** Density of type I hair cell (Spp1+) in 2 (*n* = 9), 18 (*n* = 4) and 24 (*n* = 4) month old utricular extrastriola. **(G)** Density of type I hair cell (Spp1+) in 2 (*n* = 9), 18 (*n* = 4) and 24 (*n* = 4) month old utricular striola.

To determine if the age-related VsEP alterations are due to hair cell loss, we immunostained tissues for the hair cell marker myosin 7a (Myo7a), which showed that the overall size of the sensory epithelium does not change during aging (Figures 3A,B). The utricular sensory epithelium contains a central striola surrounded by the extrastriolar region. Afferents innervating hair cells in striolar or extrastriolar regions have different spike regularities and response dynamics (Eatock and Songer, 2011). Importantly, hair cell density in both extrastriolar (Figure 3C) and striolar (Figure 3D) regions remains unchanged with aging. Consistent with previous studies (Desai et al., 2005), we observed higher hair cell density in the extrastriolar region than in the striola. Furthermore, specific labeling of type I hair cells with secreted phosphoprotein 1 antibody (Spp1, Figure 3E; Sakagami, 2000; McInturff et al., 2018) showed that their density does not change during aging either (Figures 3F,G). Given that total hair cell density and type I hair cell density are normal at 24 months, we concluded that neither type II hair cell density nor the type I/type II hair cell ratio change during aging as well. Together, these results indicate that the VsEP threshold elevation at 24 months is unlikely due to hair cell loss or alteration in the distribution of the hair cell types.

### Age-Dependent Synaptopathy in the Utricular Extrastriola

**Figure 4:**
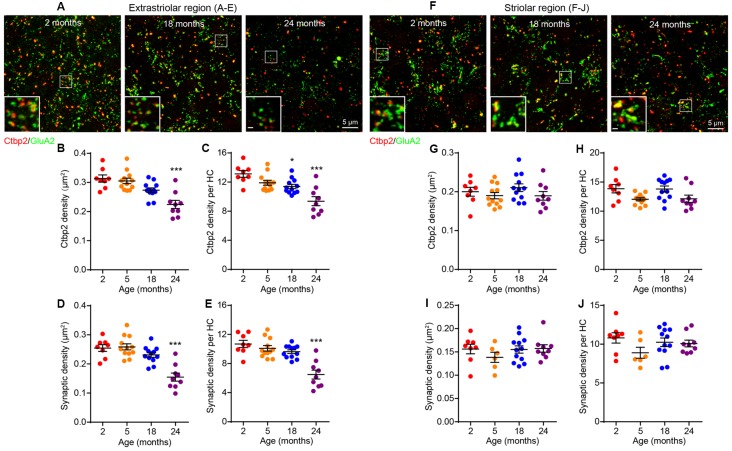
Age-related decline in utricular ribbon synapse density is restricted to the extrastriolar region and occurs only at 24 months of age.** (A–E)** Extrastriolar and **(F–J)** striolar regions of 2, 18 and 24 month old mouse utricles were analyzed separately after immunostaining with GluA2 and Ctbp2. **(A)** Representative confocal images of extrastriolar ribbon synapses. **(B,C)** Density of synaptic ribbon and **(D,E)** putative ribbon synapses either normalized to unit area **(B,D)** or each hair cell **(C,E)** in 2 (*n* = 8), 5 (*n* = 12), 18 (*n* = 12) and 24 (*n* = 9) month old utricular extrastriola. *p < 0.05 and ***p < 0.001 by one-way ANOVA comparing to 2-month-old group followed by Dunn’s post-test. **(F)** Representative confocal images of striolar ribbon synapses. **(G,H)** Density of synaptic ribbon and **(I,J)** putative ribbon synapses either normalized to unit area **(G,I)** or each hair cell **(H,J)** in 2 (*n* = 8), 5 (*n* = 6), 18 (*n* = 12) and 24 (*n* = 9) month old utricular striola.

**Figure 5:**
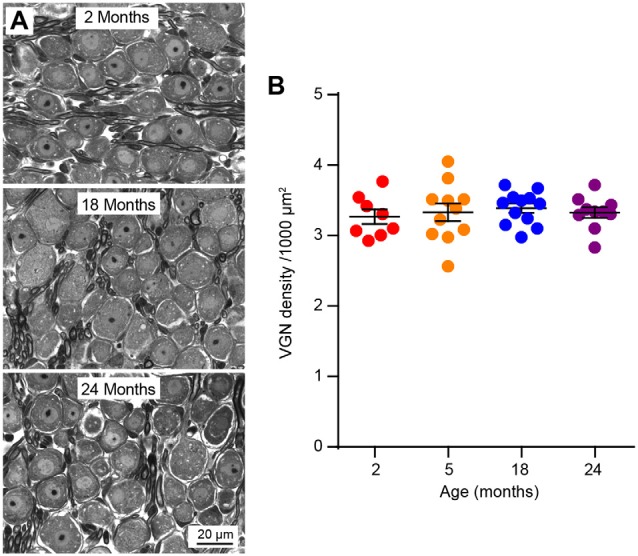
Vestibular ganglion neuron (VGN) density does not change during aging. **(A)** Representative plastic section and light microscopy images of VGN in aging mice. **(B)** VGN density in 2 (*n* = 8), 5 (*n* = 11), 18 (*n* = 12) and 24 (*n* = 10) month old mice.

The observation that age-related VsEP decline occurs in the absence of hair cell loss indicated that there might be alterations in hair cell synapses or vestibular neurons. To assess the density of hair cell ribbon synapses, we immunostained the presynaptic ribbon and postsynaptic receptor patches using antibodies against Ctbp2 and GluA2, respectively (Figures 4A,F). We quantified the number of presynaptic sites (Ctbp2+ synaptic ribbons) and putative ribbon synapses (Ctbp2-GluA2 co-localization) but not the postsynaptic sites by themselves (GluA2 alone) because their irregular morphology in the vestibular sensory epithelium (Sadeghi et al., 2014) makes quantification unreliable. We found reduced density of Ctbp2+ synaptic ribbons per unit area (Figure 4B; Kruskal–Wallis statistic = 18.63, *p* = 0.0007, one-way ANOVA followed by Dunn’s multiple comparisons test) or per hair cell (Figure 4C; Kruskal–Wallis statistic = 17.09, *p* = 0.0002, one-way ANOVA followed by Dunn’s multiple comparisons test) only at 24 months. Remarkably, this change is restricted to the extrastriolar region (Figures 4F–H). There is a slight reduction in extrastriolar presynaptic ribbon density by 18 months (Figure 4C; Kruskal–Wallis statistic = 17.09, *p* = 0.0399, one-way ANOVA followed by Dunn’s multiple comparisons test), suggesting that some animals may have begun developing utricular synaptopathy at this age, but the loss might be too mild to be reflected in VsEP defects. Similar to ribbon density, loss of putative ribbon synapses is also restricted to 24 months and to the extrastriolar region (Figures 4D,E,I,J; for Figure 4D, Kruskal–Wallis statistic = 20.28, *p* = 0.0004; for Figure 4E, Kruskal–Wallis statistic = 18.49, *p* = 0.0002; both by one-way ANOVA followed by Dunn’s multiple comparisons test). Importantly, neuronal density in the vestibular ganglion does not change during aging (Figures 5A,B), indicating that synaptopathy is not caused by loss of vestibular ganglion neurons (VGNs), and that these neurons, like hair cells (Figure 3), do not undergo significant degeneration by 24 months.

### Age-Related Synapse Loss Occurs Primarily in Extrastriolar Type I Hair Cells

**Figure 6:**
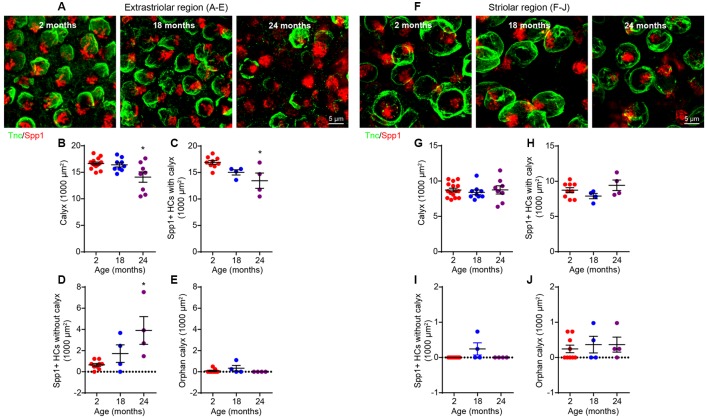
Loss of calyces in 24-month-old utricular extrastriola. **(A–E)** Extrastriolar and **(F–J)** striolar regions of 2, 18 and 24-month-old mouse utricles were analyzed separately after immunostaining with tenascin-C (Tnc) and secreted phosphoprotein 1 (Spp1). **(A)** Representative confocal images of extrastriolar calyces and type I hair cells. **(B)** Density of calyx (Tnc+) in 2 (*n* = 14), 18 (*n* = 9) and 24 (*n* = 8) month old utricular extrastriola. **(C)** Density of type I hair cell associated with calyx in 2 (*n* = 9), 18 (*n* = 4) and 24 (*n* = 4) month old utricular extrastriola. **(D)** Density of orphan type I hair cell and **(E)** density of orphan calyx in 2 (*n* = 9), 18 (*n* = 4) and 24 (*n* = 4) month old utricular extrastriola. *p < 0.05 by one-way ANOVA comparing to 2-month-old group followed by Dunn’s post-test. **(F)** Representative confocal images of striolar calyces and type I hair cells. **(G)** Density of calyx (Tnc+) in 2 (*n* = 15), 18 (*n* = 9) and 24 (*n* = 8) month old utricular striola. **(H)** Density of type I hair cell associated with calyx in 2 (*n* = 9), 18 (*n* = 4) and 24 (*n* = 4) month old utricular striola. **(I)** Density of orphan type I hair cells and **(J)** density of orphan calyx in 2 (*n* = 9), 18 (*n* = 4) and 24 (*n* = 4) month old utricular striola.

Since type I and II hair cells differ in their physiological properties (Eatock et al., 2008; Eatock and Songer, 2011), we considered it important to determine whether the synaptopathy affects both hair cell types. We first studied type I hair cells by co-staining for Spp1 (for type I cells) and tenascin C (Tnc), a marker for calyceal terminals (Swartz and Santi, 1999; Figures 6A,F). This revealed an extrastriolar-specific reduction in the density of calyceal terminals at 24 months (Figure 6B; Kruskal–Wallis statistic = 5.607, *p* = 0.0392, one-way ANOVA followed by Dunn’s multiple comparisons test), resulting in decreased density of type I hair cells associated with calyces (Figure 6C; Kruskal–Wallis statistic = 7.639, *p* = 0.0323, one-way ANOVA followed by Dunn’s multiple comparisons test) and the appearance of a significant number of “orphan” extrastriolar type I hair cells lacking calyceal innervation (Figure 6D; Kruskal–Wallis statistic = 7.831, *p* = 0.0104, one-way ANOVA followed by Dunn’s multiple comparisons test). The presence of “orphan” calyx, defined by a calyx decoupled from a hair cell, was negligible at all ages examined (Figure 6E). These changes do not occur in the striolar region (Figures 6F–J).

**Figure 7:**
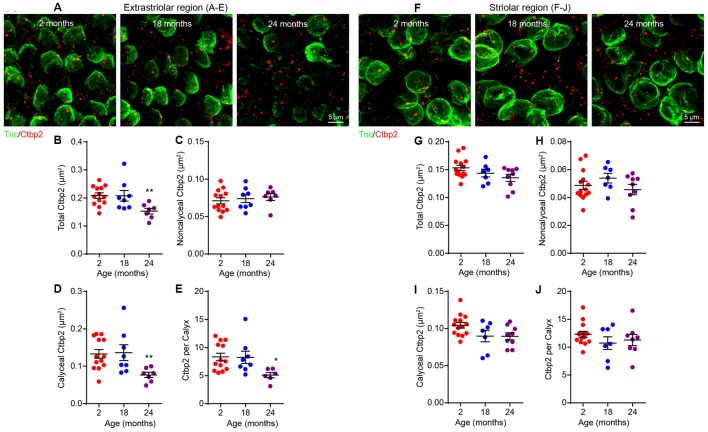
Loss of calyceal synapses but not bouton synapses in 24-month-old utricular extrastriola. **(A–E)** Extrastriolar and **(F–J)** striolar regions of 2, 18 and 24-month-old mouse utricles were analyzed separately after immunostaining with tenascin-C (Tnc) and Ctbp2. **(A)** Representative confocal images of extrastriolar calyces and synaptic ribbons. Density of total synaptic ribbon **(B)**, noncalyceal ribbon **(C)** and calyceal ribbon **(D)** in 2 (*n* = 13), 18 (*n* = 8) and 24 (*n* = 7) month old utricular extrastriola. **(E)** Number of synaptic ribbons associated with each calyx in 2 (*n* = 13), 18 (*n* = 8) and 24 (*n* = 7) month old utricular extrastriola. *p < 0.05 and **p < 0.01 by one-way ANOVA comparing to 2-month-old group followed by Dunn’s post-test. **(F)** Representative confocal images of striolar calyces and synaptic ribbons. Density of total synaptic ribbon **(G)**, noncalyceal ribbon **(H)** and calyceal ribbon in **(I)** 2 (*n* = 14), 18 (*n* = 7) and 24 (*n* = 9) month old utricular striola. **(J)** Number of synaptic ribbons associated with each calyx in 2 (*n* = 14), 18 (*n* = 7) and 24 (*n* = 9) month old utricular striola.

To test for the association between the age-related loss of both ribbon synapses (Figure 4) and calyces (Figure 6), we co-immunostained utricular sensory epithelia for Ctbp2 and Tnc (Figures 7A,F). Ctbp2 puncta associated with Tnc were classified as calyceal ribbon synapses, whereas the other Ctbp2 puncta as putative bouton-like synapses. As shown in previous samples (Figure 4), the total density of synaptic ribbons is significantly reduced at 24 months only in extrastriolar region (Figures 7B,G; Kruskal–Wallis statistic = 9.893, *p* = 0.0042, one-way ANOVA followed by Dunn’s multiple comparisons test). Remarkably, the density of Tnc-associated Ctbp2 puncta is reduced in the extrastriolar region (Figures 7D,I; Kruskal–Wallis statistic = 9.982, *p* = 0.0057, one-way ANOVA followed by Dunn’s multiple comparisons test), whereas the density of ribbon puncta not associated with calyces does not change (Figure 7C). Like synaptic density (Figures 4F–J) and calyx density (Figures 6F–J), the density of calyceal synapses is normal in the aged striola (Figures 7F–J).

**Figure 8:**
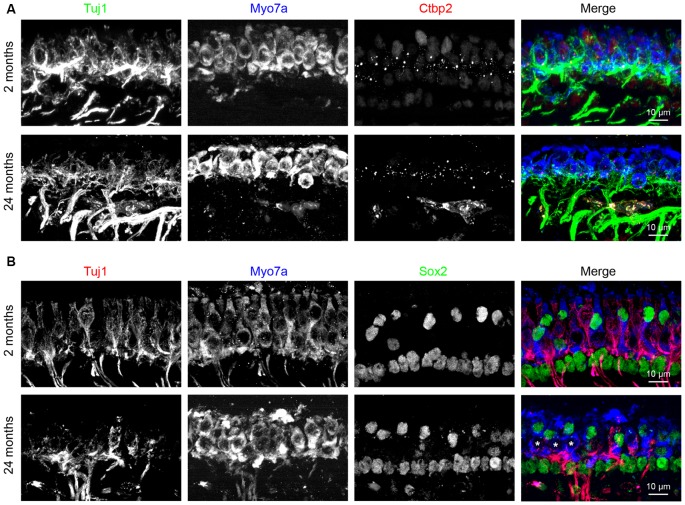
Loss of calyceal innervations and ribbons of type I hair cells in 24-month-old utricular extrastriola. **(A)** Co-labeling of calyceal terminals (Tuj1), hair cells (Myo7a) and synaptic ribbons (Ctbp2) in 2 and 24 months old utricular extrastriolar region. **(B)** Co-labeling of calyceal terminals (Tuj1), hair cells (Myo7a) and type II hair cells + supporting cells (Sox2) in 2 and 24 months old utricular extrastriolar region. Asterisks (*) indicate Sox2 type I hair cells that had lost calyceal innervations in 24-months-old utricle.

**Figure 9:**
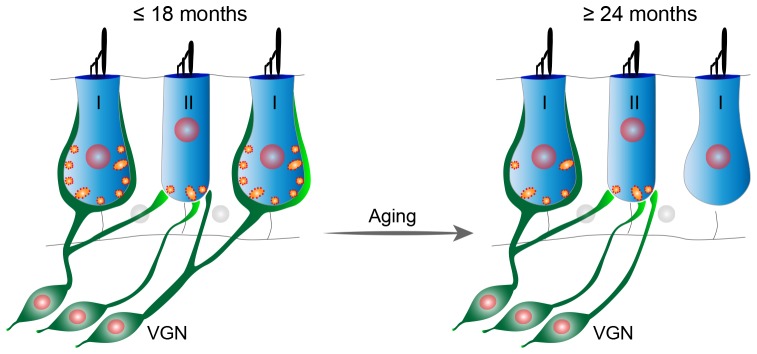
Proposed model of age-related vestibular synaptopathy in FVB/N mouse utricle. Utricular structure and function are normal until 18 months of age, but by 24 months of age there is a significant loss of calyceal nerve terminals and their associated synaptic ribbons in the extrastriolar region with a corresponding alteration in VsEPs.

To further test the notion that the age-related utricular extrastriolar synapse loss is primarily due to loss of type I hair cell calyces, we performed immunostaining analyses of cryo-sectioned samples (Figure 8). Tuj1 staining, which labels all nerve fibers and terminals, shows that calyceal terminals have normal appearance in young (2-month old)-mice (Figures 8A,B, top panels), but are disarrayed in tissues from 24-month-old mice (Figures 8A,B, bottom panels). Consistent with the synaptic loss observed in wholemounts (Figures 4, 7), Ctbp2+ synaptic ribbons are also reduced in the sectioned material derived from older mice (Figure 8A). As Sox2 is expressed by adult supporting cells and type II hair cells, but not type I hair cells (Oesterle et al., 2008; Warchol et al., 2019), type I hair cells can also be identified as Myo7a+/Sox2− cells. While all extrastriolar Myo7a+/Sox2− type I hair cells show Tuj1+ calyceal enclosures at 2 months of age, type I hair cells in the 24 months old utricle present extensive loss of calyceal innervations (Figure 8B). Together, these results indicate that calyces and calyceal synapses are specifically lost in the utricular extrastriola at 24 months, suggest that this synaptopathy might contribute to the deficits in vestibular functions in these old animals (Figure 9).

## Discussion

Our results demonstrate that age-related otolith organ dysfunction in mice begins later than age-related hearing loss. The physiological deterioration measured by VsEP correlates with loss of utricular ribbon synapses without any evidence of hair cell or neuronal loss, suggesting that synaptopathy is a key contributor to ARVD. Remarkably, the synaptic pathology is both cell type- and region-specific, i.e., loss of ribbon synapses is limited to type I hair cell calyceal innervations within the extrastriolar region.

Decline in oVEMP amplitudes and increase in latencies demonstrates age-dependent utricular dysfunction in humans (Iwasaki et al., 2008; Agrawal et al., 2012), but whether this is associated with hair cell loss remains controversial. Whereas a study based on serial sections found approximately 20% loss of utricular hair cells in subjects aged 70 years and above (Merchant et al., 2000), another study based on unbiased stereological analysis found no age-related loss of hair cells in utricles of subjects with mean age of 82 years (Gopen et al., 2003). We found no utricular hair cell loss in aged FVB/N mice, consistent with findings in humans (Gopen et al., 2003), C57BL/6J mouse (Mock et al., 2016) and Fischer 344 rats (Nakayama et al., 1994), supporting the notion that hair cell loss is not the primary cause of age-related utricular dysfunction. However, it is unclear if subtle changes in hair cells, e.g., stereocilia abnormalities or lipofuscin aggregates, might also contribute to age-related vestibular decline (Richter, 1980; Anniko, 1983).

A low level of utricular hair cell turnover has been observed in the normal mouse, with newly generated hair cells being primarily type II hair cells (Bucks et al., 2017). Thus, it seems plausible that increased number of new type II hair cells may compensate for the loss of type I hair cells, altering the ratio of type I/II hair cells without total hair cell loss. However, we did not observe a decrease in the density of type I hair cells or a change in the ratio type I/type II cells, consistent with the notion that hair cell replacement is relatively rare in normal adult utricle and it plays a negligible role in utricular aging.

VGN density remains stable from 2 to 24 months, consistent with previous reports of normal VGN numbers in 32-month-old C57BL/6J mice (Cohen et al., 1990) and 31-month-old rats (Alidina and Lyon, 1990; Lyon and King, 1997). A similar discordance between loss of function and neurons was reported for aged and noise-exposed cochleae (Kujawa and Liberman, 2009; Sergeyenko et al., 2013), indicating that loss of sensory neurons is not the initial contributor of age-related inner ear dysfunction in the mouse. Whether this is the case in humans remains controversial since some studies have shown age-dependent decline in total VGN numbers (Richter, 1980; Velázquez-Villaseñor et al., 2000; Park et al., 2001) while others have found no change in the number of total vestibular nerve fibers (Rasmussen, 1940; Moriyama et al., 2007). Thus, further studies will be needed to determine how aging alters human VGNs.

A previous study reported a linear relationship between aging and changes in VsEP threshold, P1 amplitude and latency in C57BL/6J mice (Mock et al., 2016). In contrast, we found that the effects of aging on otolith organ function are non-linear in FVB/N mice, with defects starting between 18 and 24 months of age. As the animals used by Mock et al. (2016) were mostly 21 months and younger and were not in discreet age groups, it is possible that the power of the experiment might not have been sufficient to detect the most dramatic deterioration in utricular function. Furthermore, despite observing age-related utricular function impairments, Mock et al. (2016) did not to detect changes in hair cell or synapse density. It is possible that age-related synapse loss was missed since the study did not differentiate between striolar and extrastriolar synapses.

Vestibular hair cells are heterogeneous in their location (striola vs. extrastriola) and innervation patterns (synaptic bouton vs. calyx). Such heterogeneity is responsible for diversification of afferent physiology (Eatock and Songer, 2011). In the extrastriolar region, type I hair cells are innervated by calyceal-terminals of dimorphic afferent neurons (innervating both type I and type II hair cells), while the type II hair cells form synapses with either the bouton-only afferents or bouton-terminals of the dimorphic afferents (Figure 9, left). Based on the observations that both calyx density (Figure 6B) and calyceal synapse density (Figure 7D) are reduced, while the non-calyceal (bouton) synapses remain unchanged (Figure 7C), it is likely that the calyceal branches of dimorphic afferents are specifically impaired during aging (Figure 9).

The functional implications of extrastriolar calyceal synapse loss may be 2-fold. First, extrastriolar afferents in mammals display strikingly regular stimulus-evoked spike timing, while the striolar afferents show highly irregular timing (Baird et al., 1988; Goldberg et al., 1990; Sadeghi et al., 2007). An important feature of the regular extrastriolar afferents is that they have less adapting response dynamics with tonic and low-pass filtering characteristics, enabling them to signal steady-state changes in head orientation relative to gravity and make them more sensitive to motion transients (Goldberg, 2000). Therefore, loss of these regular afferents may impair the spike-time coding of the motion changes. Interestingly, microgravity during spaceflight also results in specific loss of utricular extrastriolar synapses in mice (Sultemeier et al., 2017), highlighting a potential similarity between vestibular aging and vestibular sensory deprivation. Second, in contrast to bouton afferents, in addition to the quantal release of ribbon synaptic vesicles that produces large and rapid EPSCs, calyceal terminals can transmit non-quantal mechanically evoked signals (Yamashita and Ohmori, 1990). Non-quantal transmission, presumably achieved by accumulation of potassium (Goldberg, 1996; Lim et al., 2011; Contini et al., 2017), glutamate (Songer and Eatock, 2013; Sadeghi et al., 2014) or proton (Highstein et al., 2014) at the thin synaptic cleft and direct depolarization may avoid delays in the processes of conventional neurotransmitter signaling. During fast head motions, the calyceal synapses can adjust reflex responses rapidly owing to the boost from non-quantal transmission. Thus, specific deterioration of calyceal synapses may impair the timeliness of response to change in head motions, leading to increased problems with balance in aged animals.

We observed different onsets for age-related cochlear and vestibular otolith organ dysfunction in FVB/N mice, with hearing loss starting at 5 months and vestibular impairment being apparent only at 24 months of age. Previous studies with C57BL/6J and CBA/CaJ mice have shown strain difference in the progression of vestibular otolith organ aging, i.e., two studies found that VsEP degradation onsets later than hearing loss in C57BL/6J mice (Shiga et al., 2005; Mock et al., 2016) while another showed they occur at similar rates in CBA/CaJ mice (Mock et al., 2011). The genetic determinant of these strain differences is currently unknown, but the possible role of strain-specific Cdh23 mutation in C57BL/6J has been ruled out in a previous study (Mock et al., 2016). A recent report indicated that IHCs in C57BL/6J mice have larger Ca^2+^ currents, release more synaptic vesicles and recycle synaptic vesicles more rapidly than those in CBA/CaJ mice (Liu et al., 2019). If vestibular hairs cells in C57BL/6J mice also have similar characteristics, it could explain some of the reported strain differences in vestibular aging and would suggest that excessive exocytosis and glutamate toxicity plays an important role in this process. More importantly, a faster age-dependent decrease in auditory responses is also observed in a longitudinal study recording both auditory and vestibular functions in healthy older human subjects (Enrietto et al., 1999). These observations indicate that the late-onset of vestibular dysfunction may be a common phenomenon in the aging mammalian inner ear, and our findings from FVB/N mice recapitulate the progression of vestibular disorder in humans.

Our results and those of Sergeyenko et al. (2013) indicate that the onsets of cochlear and vestibular dysfunction are closely correlated with synaptic degeneration in their respective sensory epithelia. The molecular mechanism of age-related synapse loss is not well understood; however, it is speculated that continuous stimulation of the sensory system generates excitotoxicity that accumulates and eventually causes synaptic damage (Pujol et al., 1993; Wan and Corfas, 2015). Thus, the differences in tissue responses to excitotoxicity may explain the distinct vulnerabilities of cochlear and vestibular synapses. During neural transmission, excess glutamate at the synaptic cleft is taken up by glutamate transporters, including EAATs (O’Shea, 2002). Supporting cells surrounding the IHCs express EAAT-1 (GLAST; Furness and Lehre, 1997), and EAAT-1 knockout increases noise-induced hearing loss (Hakuba et al., 2000). In addition to EAAT-1, EAAT-4 and EAAT-5 are also expressed in the vestibular sensory epithelia (Dalet et al., 2012; Schraven et al., 2012), which may provide additional mechanism to suppress glutamate excitotoxicity in the vestibular system. Interestingly, calcium permeable AMPARs lacking GluA2 subunit have been reported in ribbon synaptic terminals in zebrafish and bullfrog, and immunostaining suggests that such channels are present in IHC synapses (Sebe et al., 2017). If these types of channels are present in the vestibular synapses and their expression changes during aging, they could contribute to increased excitotoxicity and synaptic degeneration in the aging utricle, but this possibility remains to be tested.

The age-related decrease in ABR peak 1 amplitude we found in FVB/N mice is similar to the one previously observed in CBA/CaJ mice (Sergeyenko et al., 2013). That study provided strong evidence that IHC synapse loss is the most likely contributor to the functional decline. Thus, it is likely that synapse loss contributes to the ABR decline in the FVB/N strain. However, in our mice, we also found a progressive increase in ABR peak 1 latencies. Previous studies showed that noise-induced synaptopathy does not lead to increased latency (Wan and Corfas, 2017), but we showed that auditory nerve demyelination causes both decrease in ABR peak 1 amplitude and increase latency (Wan and Corfas, 2017). Thus, our current observations support the possibility that in some animal models, demyelination might contribute to age-related hearing loss and vestibular dysfunction by altering action potential timing, firing latency and synchronization (Xing et al., 2012; Kim et al., 2013; Wan and Corfas, 2017).

Together with the early-onset synaptopathy previously observed in aging cochlea (Sergeyenko et al., 2013), our study indicates that synaptic loss is one of the earliest and possibly major contributor to both age-related hearing and vestibular impairment. Currently, no treatments are available for balance disorders except for physiotherapy to aid central compensation. We previously reported that the neurotrophic factors Bdnf and Ntf3 are required for ribbon synapse formation and maintenance in the vestibular and cochlear sensory epithelia respectively (Gómez-Casati et al., 2010; Wan et al., 2014). Importantly, overexpression or round window application of Ntf3 promotes the cochlear ribbon synapse regeneration and hearing recovery after noise overexposure (Wan et al., 2014; Suzuki et al., 2016). As the expression of Bdnf and Ntf3 decreases with age in the inner ear (Rüttiger et al., 2007; Sugawara et al., 2007), it is conceivable that neurotrophic factors could be used to treat age-related hearing loss and vestibular disorders.

## Data Availability

All datasets generated for this study are included in the manuscript.

## Ethics Statement

All animal procedures were approved by the Institutional Animal Care and Use Committee of the University of Michigan.

## Author Contributions

GW and GC designed the study. GW, LJ, TS, SG and G-PW performed the experiments and analyzed the data. GW and GC interpreted the data and wrote the manuscript with the help of other authors.

## Conflict of Interest Statement

GC is a scientific founder of Decibel Therapeutics, has an equity interest in and has received compensation for consulting. The company was not involved in this study. The remaining authors declare that the research was conducted in the absence of any commercial or financial relationships that could be construed as a potential conflict of interest.

## Footnotes

^1^https://www.cdc.gov/media/releases/2016/p0922-older-adult-falls.html
